# Insomnia and depression as risk factors for dementia. A scoping review

**DOI:** 10.1192/j.eurpsy.2023.253

**Published:** 2023-07-19

**Authors:** I. Duran-Cristobal, A. Noguero-Alegre, A. M. Matas-Ochoa, S. Rubio-Corgo, F. J. Gomez-Beteta

**Affiliations:** Psychiatry, Infanta Leonor University Hospital, Madrid, Spain

## Abstract

**Introduction:**

One of the most important functions of sleep may be the promotion of brain development. The non-REM and REM sleep sequences show the succession of cerebral processing phenomena that underlie memory consolidation. The negative consequences of sleep loss on neural and behavioral plasticity has been examined. On the other hand, sleep disruption can be a crucial symptom to develop depression disorders. Recent literature suggests that maintenance insomnia may be a risk factor for dementia. It would be important to elucidate which factors may increase the risk of developing dementia and aggravating its progression.

**Objectives:**

The aim of this scoping review is to point out the increased risk of developing dementia related to insomnia and depression.

**Methods:**

Relevant literature was searched with PUBMED as electronic database. We used and combined the following MeSH terms: depression, insomnia, cognitive impairment and dementia. We chose sixteen recent studies from 2009 to 2021. Four of them were ruled out because the methodology and conclusions were not enough evident.

**Results:**

We underlined an interesting research which was carried out with Chinese population in 2021. A total of 256 patients with insomnia disorder were diagnosed by neurologists, 45 of whom were diagnosed with amnesic mild cognitive impairment (aMCI) and 45 participants with intact cognition were chosen as controls matched for age and education. A case-control study was conducted to compare sleep structure between aMCI and control patients with insomnia disorder. An American prospective research in 2016 founded a statistically significant association with a higher MCI/dementia risk in women with either short (≤6 hours/night) or long (≥8 hours/night) sleep duration (vs.7 hours/night). The relationships between depression, cognitive function, serum brain-derived neurotrophic factor (BDNF) and volumetric MRI measurements in older adults were investigated. A total of 4352 individuals aged 65 years or older (mean age 72 years) participated in this Japanese study.

**Conclusions:**

According to these researches, we emphasize the importance of detecting sleep disturbances as potential risk factors for MCI and dementia. All of them provide evidences that future studies should investigate dementia prevention among elderly individuals through the management of insomnia. At that point we have to consider personalized medicine and machine learning techniques for sleep and cognitive or mood symptoms.

**Disclosure of Interest:**

None Declared

